# Design of SARS-CoV-2 Mpro, PLpro dual-target inhibitors based on deep reinforcement learning and virtual screening

**DOI:** 10.4155/fmc-2021-0269

**Published:** 2022-02-27

**Authors:** Li-chuan Zhang, Hui-lin Zhao, Jin Liu, Lei He, Ri-lei Yu, Cong-min Kang

**Affiliations:** ^1^College of Chemical Engineering, Qingdao University of Science & Technology, Qingdao, 266042, China; ^2^Key Laboratory of Marine Drugs, Chinese Ministry of Education, School of Medicine & Pharmacy, Ocean University of China, Qingdao, 266003, China

**Keywords:** covalent docking, deep reinforcement learning, drug design, molecular dynamics simulation, Mpro, PLpro

## Abstract

**Background:** Since December 2019, SARS-CoV-2 has continued to spread rapidly around the world. The effective drugs may provide a long-term strategy to combat this virus. The main protease (Mpro) and papain-like protease (PLpro) are two important targets for the inhibition of SARS-CoV-2 virus replication and proliferation. **Materials & methods:** In this study, deep reinforcement learning, covalent docking and molecular dynamics simulations were used to identify novel compounds that have the potential to inhibit both Mpro and PLpro. **Results and conclusion:** Three compounds were identified that can effectively occupy the Mpro protein cavity with the PLpro protein cavity and form high frequency contacts with key amino acid residues (Mpro: His41, Cys145, Glu166, PLpro: Cys111). These three compounds can be further investigated as potential lead compounds for SARS-CoV-2 inhibitors.

In this century, three globally endemic coronaviruses have emerged: the severe acute respiratory syndrome coronavirus (SARS-CoV), the Middle East respiratory syndrome coronavirus (MERS-CoV) and the severe acute respiratory syndrome coronavirus 2 (SARS-CoV-2). SARS-CoV-2 emerged in Wuhan, China, in December 2019 [1]. Up to the beginning of May 2021, COVID-19 had spread to hundreds of countries, infecting more than 150 million people and causing more than three million deaths; the virus is still spreading on a large scale in the world. *De novo* drug design can provide effective treatment for COVID-19. It also has meaningful impact on the prevention and treatment of similar viruses in the future.

SARS-CoV-2 is an enveloped, positive-sense, single-stranded RNA virus [2]. When it enters the cell, it translates its genetic information into two polyprotein 1a/1ab (pp1a/pp1ab), which mediate viral replication and transcription, and translates into 16 nonstructural proteins (nsp1–nsp16) by viral proteases [3]. This is the key step for virus replication [4]. The two critical proteases are main protease (Mpro) and papain-like protease (PLpro). PLpro is responsible for the proteolytic cleavage of nsp1–3; all the others, from nsp4 to nsp16, are cleaved by Mpro. SARS-CoV-2 is susceptible to induced mutations, whereas Mpro and PLpro are highly conserved because mutations in key proteins are usually fatal to viruses. Therefore, drugs targeted to the conserved Mpro and PLpro can prevent virus from replication and proliferation and have broad-spectrum antiviral activity. In addition, drugs that inhibit Mpro and PLpro can also reduce the risk of drug resistance associated with viral mutations [5]. Previous studies have demonstrated that peptide inhibitors of Mpro and PLpro have significantly different substrate specificities [6]. Therefore, it is not easy to design a peptide inhibitor that can act on these two proteases. In this situation, design of small molecule inhibitors that can inhibit both SARS-CoV-2 Mpro and PLpro is a valuable goal.

The Mpro active site contains a catalytic dyad consisting of the conserved residues His41 and Cys145, and the PLpro active site contains a catalytic triad consisting of Cys111, His272 and Asp286 [7]. The fact that cysteine in both active sites plays a pivotal role in virus replication makes it possible to design dual-target covalent inhibitors. Unlike conventional drugs, covalent inhibitors have high affinity to their targets and high biological activity; however, if off-target, this high affinity can also act on other proteins, which usually leads to adverse effects [8]. The specific toxicity of covalent inhibitors has been debated for years, but studies have shown that the ideal covalent inhibitors have relatively high selectivity and low off-target interactions. This indicates that highly selective covalent inhibitors designed for viral proteins will have high biological activity and few adverse effects.

In recent years, deep learning (DL) has been applied to various aspects of drug development, such as constructing a quantitative structure activity relationship and predicting compound properties (e.g., toxicity, partition coefficient and affinity for specific target). In addition to these, DL is also used for chemical structure generation. There are several approaches to molecular generation, such as character-level recurrent neural networks [9,10], variational autoencoders [11–13], adversarial autoencoders [14], junction tree variational autoencoders [15], LatentGAN [16] and graph neural networks [17]. The current models mostly generate 1D or 2D molecules, with less exploration of generating 3D molecules. Although recently proposed neural networks such as L-Net [18] and ConfVAE [19] represent good attempts for generating 3D molecules, generating small molecules with activity directly for the target binding site remains a challenge. DL has unique advantages in acquiring small molecule features and balancing the effectiveness between targets, which can help medicinal chemists design multitargeted small molecules.

In this study, the dual-target covalent inhibitors of Mpro/PLpro were designed using deep reinforcement learning (RL) and traditional computer-aided drug design methods. A library of Mpro/PLpro small molecules was generated using deep RL. The molecules were then screened using molecular docking and dynamics simulations to obtain covalent inhibitors that act on both targets (workflow is shown in Figure 1). In this way, we expect to get the lead compound and ultimately obtain highly effective and low-toxicity small molecule drugs to treat SARS-CoV-2, which should have broad-spectrum antiviral activity and be effective on mutated viruses.

**Figure 1.**
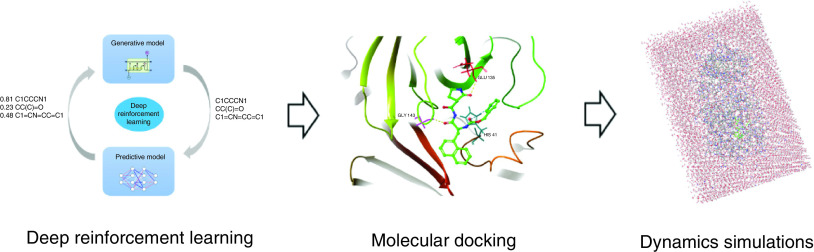
The workflow of experiment design.

## Materials & methods

### Data curation

For the pretraining set, approximately 800,000 SMILES strings of druglike molecules were collected from the ChEMBL database [20]. Molecules with molecular weightsthat were too high or too low and molecules containing uncommon groups were removed from the SMILES strings. The molecules of the fine-tuning set were obtained from ChEMBL [20], Protein Data Bank [21] and the literature [22–29]. Considering that the protease of SARS-CoV-2 is highly homologous to SARS-CoV (Mpro: 96%, PLpro: 83%), SARS-CoV inhibitors are also potent against SARS-CoV-2. To enlarge the fine-tuning set, small molecule compounds that inhibited SARS-CoV and SARS-CoV-2 were collected. Finally, the ChEMBL dataset contained approximately 800,000 compounds and the fine-tuning set contained 463 compounds. These two datasets were used to train the generative model.

High plasma concentrations of the drug may inhibit essential enzymes and cause adverse reactions, and an IC_50_ of <10 μM is usually acceptable [30,31]. For this reason, the compounds in the fine-tuning set were separated into two groups based on activity: compounds with IC_50_ <10 μM were labeled as active samples, and the rest were labeled as inactive samples. In consideration of the importance of inactive data in DL for drug design, the inactive dataset was expanded with the compounds with noninhibition to the SARS-CoV-2 virus [32]. Finally, the Mpro dataset contained 383 positive and 1631 negative samples, and that of PLpro dataset contained 70 positive and 1423 negative samples. These datasets were used to construct the predictive models.

### Model construction

The long short-term memory neural network model (LSTM) was used as the generative model. The model was constructed using the *de novo* drug design artificial intelligence tool REINVENT 2.0 [33]. First, the model was pretrained with 200 epochs using the ChEMBL dataset to ensure that it could generate the molecule with the correct chemical structure, then the model was fine-tuned with 50 epochs using the fine-tuning set. The construction of the generative model was referenced to the example in ReinventCommunity (https://github.com/MolecularAI/ReinventCommunity) and related references [34–36]. The LSTM model used the default parameters from the example.

The Random Forest classifier [37], generated by scikit-learn [38], was used as the prediction model, with n_estimators set to 100 and class_weight set to balanced. The input data to the model were the ECFP6 fingerprints [39] with 2048 bits calculated by the RDKit [40] fingerprinting algorithm. Each molecule in the dataset was transformed into a 2048D vector. Afterward, the predictive power of the model was tested using a fivefold cross-validation approach.

### Reinforcement learning

The generative and predictive models were integrated into the RL system. Four indicators were used to evaluate the small molecules: the activities to Mpro and PLpro, synthetic accessibility score [41] (SA) and quantitative estimate of drug-likeness [42] (QED). The forecasting weights of Mpro and PLpro activities were both set to four and that of SA and QED were both set to one. Compounds with the final total score >0.6 were recorded. A total of 3000 epochs were trained. A total of 4428 small molecules were obtained.

### Molecular docking

Covalent docking was performed on the covalent docking module (CovDock) of the Schrödinger Suite [43]. The crystal structures of SARS-CoV-2 Mpro (Protein Data Bank [PDB] ID: 7K40) and SARS-CoV-2 PLpro (PDB ID: 7JN2) were downloaded from the PDB (https://www.rcsb.org). Proteins were preprocessed with the Protein Preparation Wizard [44] by assigning bond orders, adding hydrogen and performing restrained energy minimization of the added hydrogen using the OPLS_2005 forcefield [45]. The previously obtained compounds were first processed using the LigPrep module and then further filtered using the Ligand Filtering module; reaction type was set to nucleophilic addition to a double bond. The grid box was a cube with 20-Å sides centered on selected amino acid residues (Mpro: Cys145; PLpro: Cys111). The reaction type was set to nucleophilic addition to a double bond and the reaction residues were Cys145 of Mpro and Cys111 of PLpro. The covalent docking process started with the docking of all ligands using Virtual screening mode, followed by advanced docking of the top 20 compounds using Pose prediction mode. The MM-GBSA [46] score was used simultaneously when docking using the Pose prediction mode. Ultimately, 105 small molecules were obtained that were able to bind covalently to Mpro and PLpro.

### Molecular dynamics simulations

The molecular dynamics simulations were carried out using the Desmond program in the Schrödinger Suite with the OPLS 2005 forcefield [45,47]. The results of the covalent docking were set as the initial structure. Each complex was placed in an orthorhombic box delimited by at least 10 Å from any atom of the protein. The box was then filled with TIP3P water molecules [48]. In addition, using the default protocol, all systems were relaxed and energy minimized at 310 K and 1 atmosphere with NPT integration. Finally, 100-ns simulations were performed on well-balanced systems.

## Results & discussion

### Model evaluation & molecular generation results

**Figure 2.**
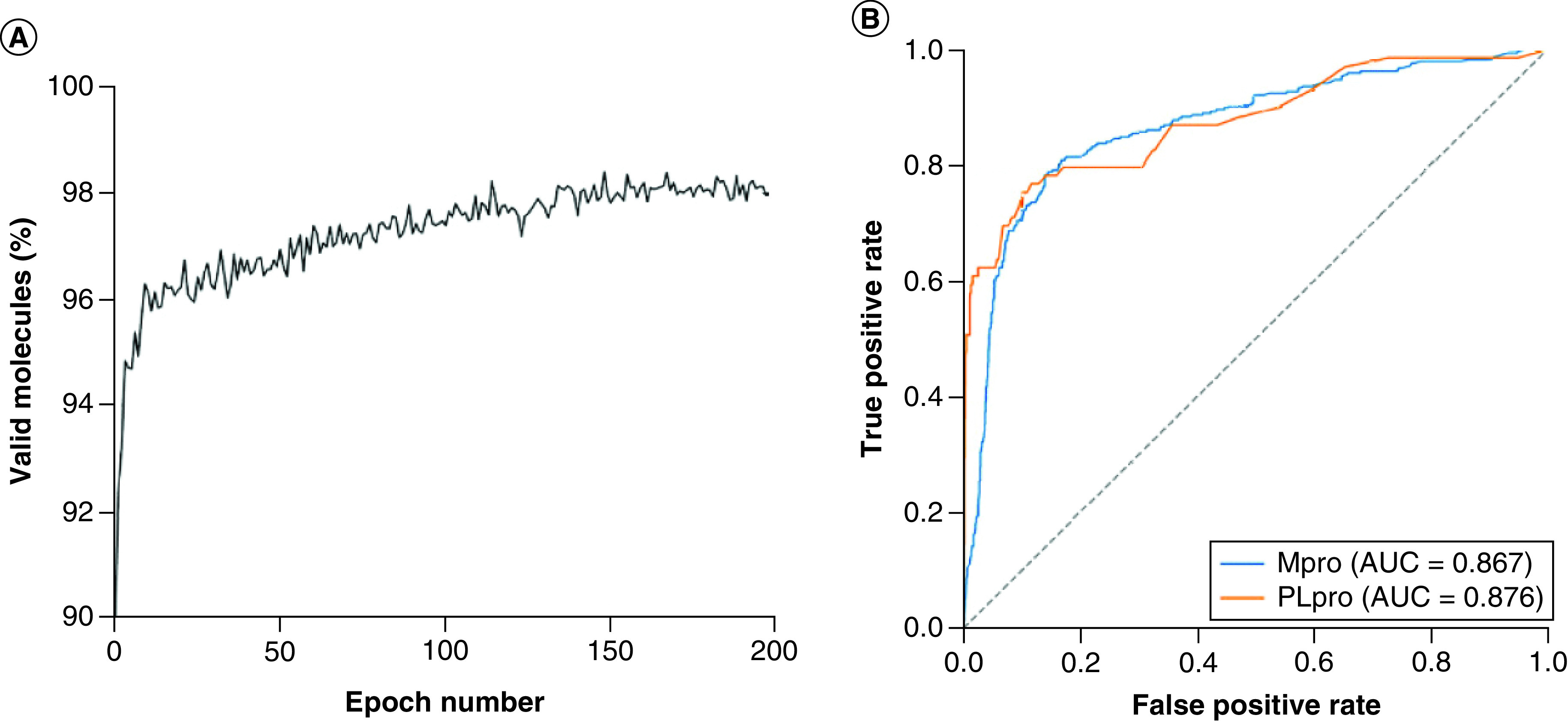
Evaluation of generative and predictive models. **(A)** The validity of the pretrained generative model. **(B)** Area under the receiver operating curves for Mpro and PLpro prediction models. AUC: Area under the curve; Mpro: Main protease; PLpro: Papain-like protease.

The generative model was constructed using REINVENT 2.0 and the predictive model was constructed using scikit-learn. The generative and predictive models were evaluated, respectively. The validity of the generated molecules was a key metric for evaluating the generative model. The result is shown in Figure 2A. After 200 epochs, an average of 98.05% valid SMILES strings were produced. This indicated that the generative model is capable to produce effective molecules. Predictive models were evaluated by fivefold cross-validation. The results are shown in Figure 2B. The area under the receiver operating curve (AUC) for the fivefold cross-validation of the Mpro and PLpro prediction models was 0.867 and 0.876, respectively; therefore, the predictive models were able to distinguish active and inactive compounds conveniently and exactly.

**Figure 3.**
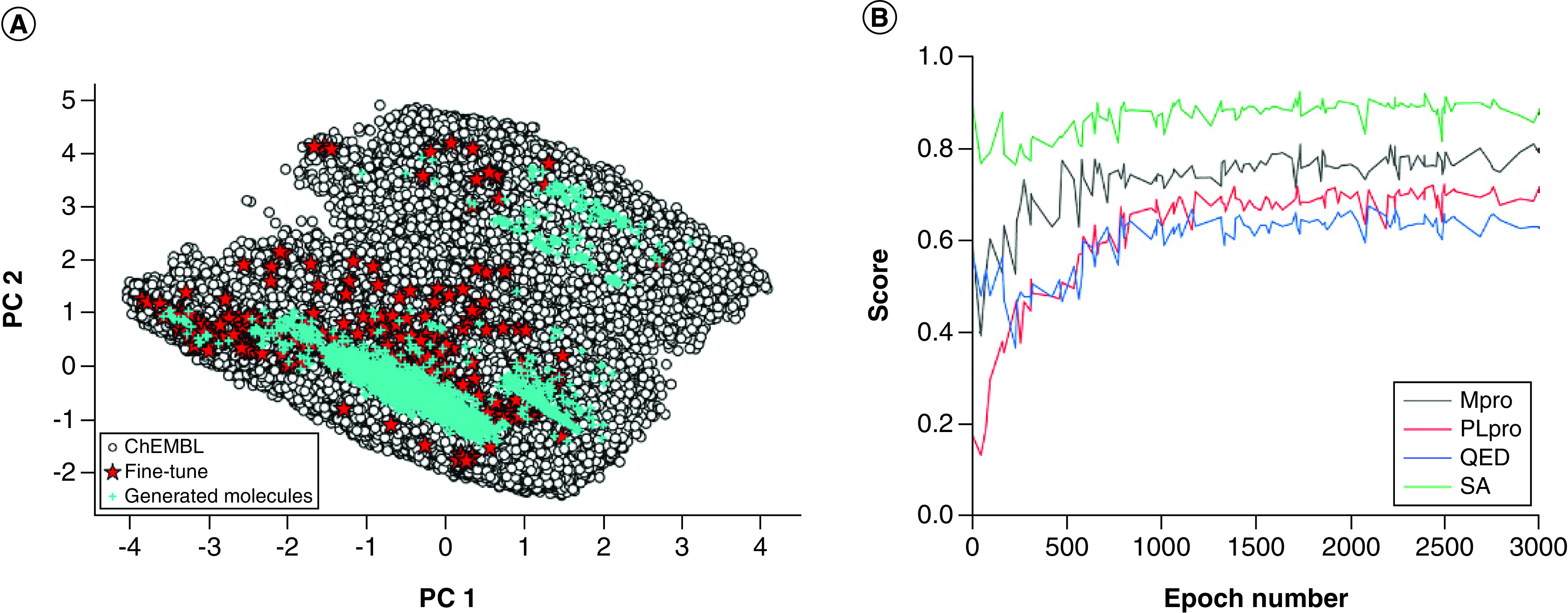
Evaluation of the generated molecules. **(A)** A principal component analysis was performed on 24 physicochemical features calculated on the pretraining set, the fine-tuned set and the generated molecular set. The first two principal components (PC1, PC2) were selected. **(B)** Scoring of molecules generated by reinforcement learning. Mpro: Main protease; PLpro: Papain-like protease; QED: Quantitative estimate of drug-likeness; SA: Synthetic accessibility score.

Subsequently, the small molecules generated by reinforcement learning were evaluated. Principal component analysis on the features of the training data and the generated small molecules (GSMs) were performed. As shown in Figure 3A, the training set, the fine-tuned set and the GSM set have similar distribution in chemical space. The chemical space of the GSMs partially overlaps with the chemical space of the training set and the fine-tuned set. The GSMs inherited the features of the small molecules in the pretrained and fine-tuned sets.

**Figure 4.**
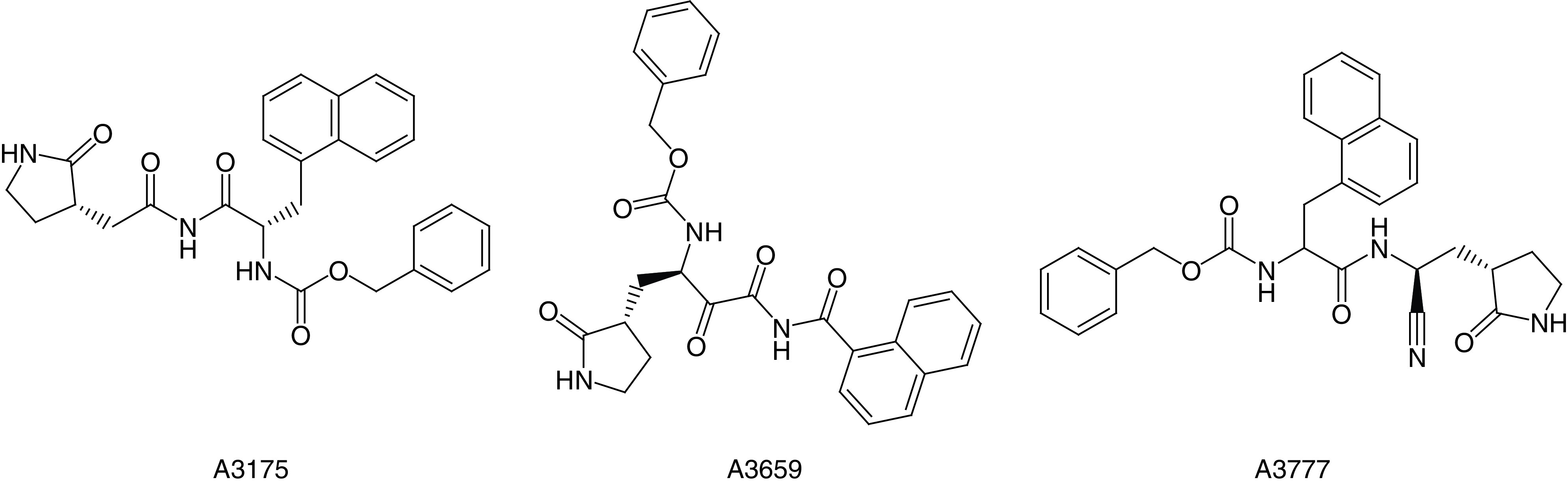
The structures of the top three compounds.

As shown in Figure 3B, after ~1000 epochs, all four scores become smooth. It can be seen that the predicted scores of Mpro activity, PLpro activity, QED and SA were stable at 0.80, 0.72, 0.65 and 0.90, respectively. Ultimately, 4428 compounds were obtained. The scores of three representative compounds are shown in Table 1, and the corresponding structures are shown in Figure 4.

**Table 1.** Scores of three representative compounds.

No.	Mpro	PLpro	SA	QED	Total
A3175	0.836	0.559	0.740	0.466	0.663
A3659	0.872	0.558	0.500	0.419	0.641
A3777	0.837	0.548	0.560	0.635	0.635

Mpro: Main protease; PLpro: Papain-like protease; QED: Quantitative estimate of drug-likeness; SA: Synthetic accessibility score.

### Molecular docking results

After covalently docked to Mpro and PLpro, 105 small molecules were obtained from the 4428 compounds. These 105 small molecules have the capacity to form covalent bonds with Cys145 of Mpro and Cys111 of PLpro. Then the top 20 compounds were docked to Mpro and PLpro using the pose prediction mode. The structures of these 20 compounds are shown in Supplementary Figure 1, and the cdock affinity scores and Molecular Mechanics Poisson-Boltzmann Surface Area (MMGBSA) scores are shown in Supplementary Table 1. The cdock affinity reflects the difficulty of forming covalent bonds between the ligand and the receptor; ligands with low scores are less likely to form covalent bonds to the receptor. Three compounds were selected by comprehensively considering the cdock affinity scores and MMGBSA scores of these 20 compounds in both targets. As shown in Table 2, all three compounds have cdock affinity scores of approximately -8 kcal/mol with Mpro, and all three compounds have cdock affinity scores below -5 kcal/mol with PLpro. This suggests that the three compounds can efficiently react with cysteine residues in the active site to form covalent bonds. Otherwise, the MMGBSA binding energies of all three compounds were below -70 kcal/mol for Mpro and -50 kcal/mol for PLpro. This indicates that all three compounds have a strong binding affinity for Mpro and PLpro.

**Table 2.** Docking scores and MM-GBSA scores of the top three compounds that bind the two proteins (kcal/mol).

Compound ID	Mpro			PLpro		
	Docking score^†^	cdock affinity^‡^	MMGBSA dG bind	Docking score^†^	cdock affinity^‡^	MMGBSA dG Bind
A3175	-8.255	-8.366	-77.73	-5.252	-5.677	-59.86
A3659	-8.991	-8.173	-78.82	-5.676	-5.504	-51.23
A3777	-8.479	-7.918	-78.70	-6.816	-6.204	-60.30

† The docking score is obtained from the covalent docking module (virtual screening mode).

‡ The cdock affinity is obtained from the covalent docking module (pose prediction mode).

MMGBSA: Molecular mechanics poisson-boltzmann surface area; Mpro: Main protease; PLpro: Papain-like protease.

**Figure 5.**
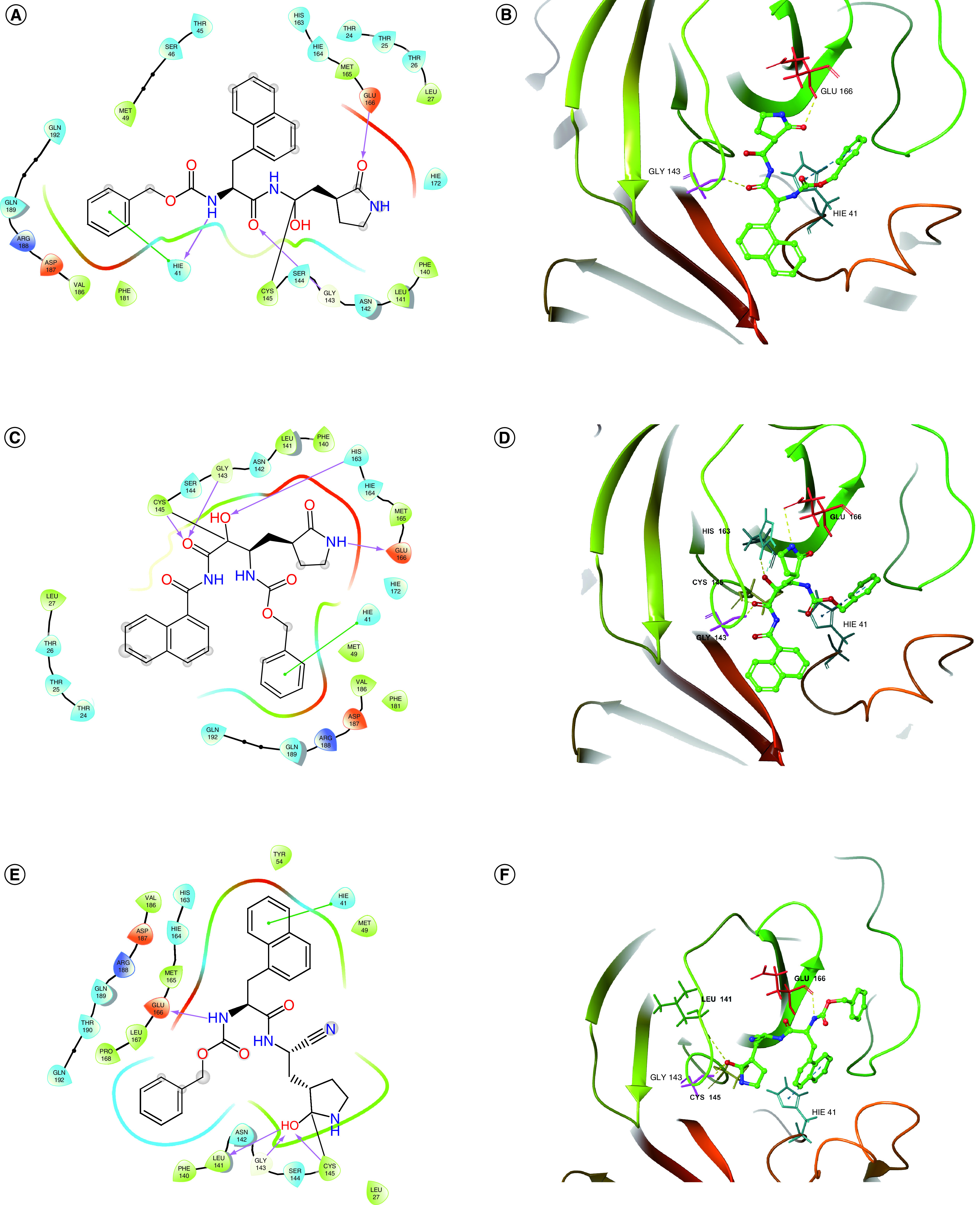
Molecular docking diagrams of the three selected compounds with main protease protein. **(A)** A 2D interaction diagram of A3175-main protease (Mpro). **(B)** A 3D interaction diagram of A3175-Mpro. **(C)** A 2D interaction diagram of A3659-Mpro. **(D)** A 3D interaction diagram of A3659-Mpro. **(E)** A 2D interaction diagram of A3777-Mpro. **(F)** A 3D interaction diagram of A3777-Mpro.

**Figure 6.**
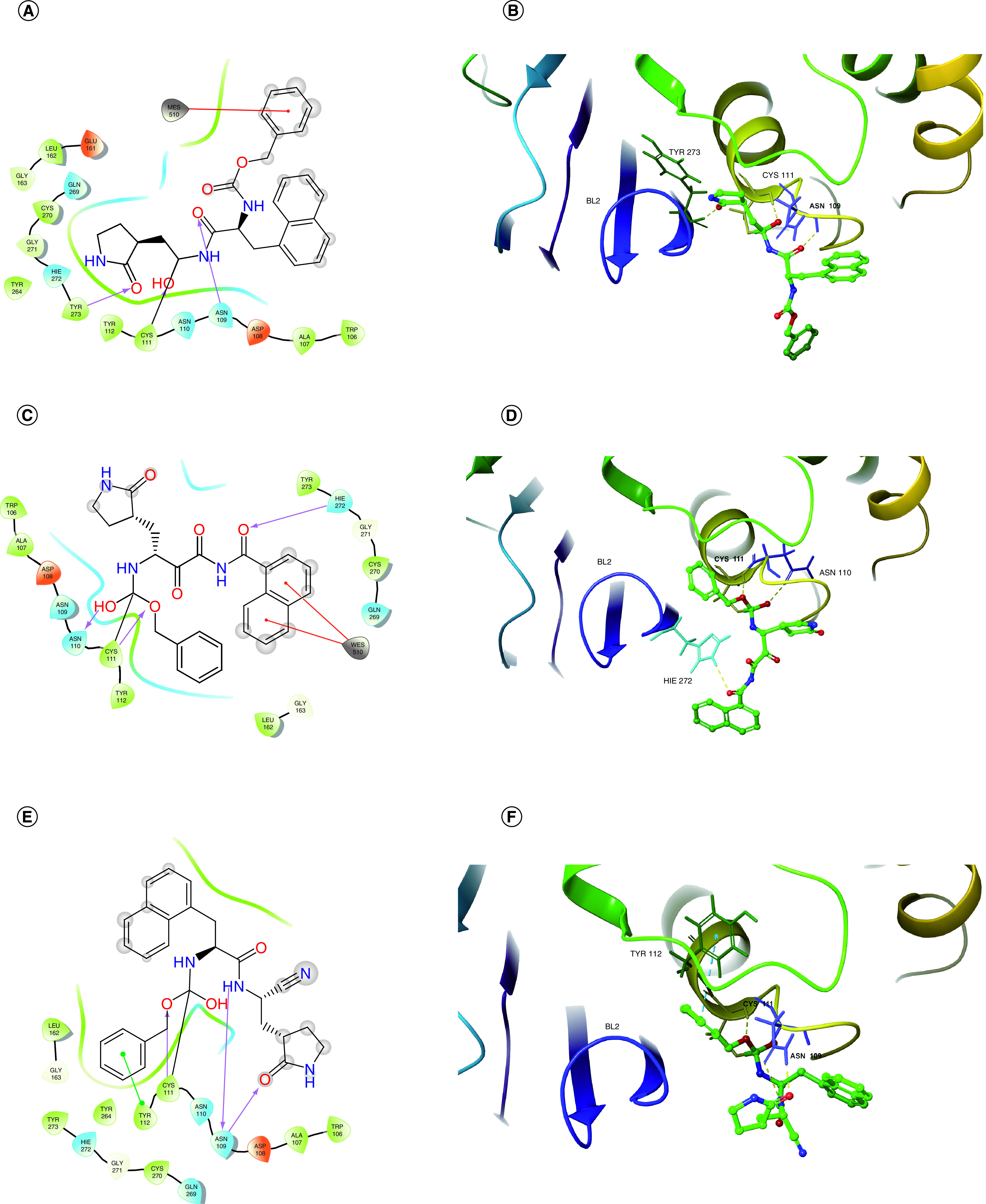
Molecular docking diagrams of the three selected compounds with papain-like protease protein. **(A)** A 2D interaction diagram of A3175-papain-like protease (PLpro). **(B)** A 3D interaction diagram of A3175-PLpro. **(C)** A 2D interaction diagram of A3659-PLpro. **(D)** A 3D interaction diagram of A3659-PLpro. **(E)** A 2D interaction diagram of A3777-PLpro. **(F)** A 3D interaction diagram of A3777-PLpro.

The molecular docking diagrams of the three compounds with Mpro and PLpro are shown in Figures 5 and 6, respectively. It can be seen that A3175, A3659 and A3777 all form covalent bonds with Cys145 of Mpro and with Cys111 of PLpro. Moreover, there are also nonbonded interactions between ligands and proteins. For Mpro, compounds A3659 and A3777 both form hydrogen bonding interactions with Cys145 and π–π interactions with His41, and compound A3175 forms hydrogen bonding interaction and π–π interaction with His41. In addition, the three compounds also form interactions with other amino acid residues within Mpro cavity, such as Leu141, Gly143, His163 and Glu166. The hydrogen bonding interaction of the ligand with Glu166 is important because Glu166 is responsible for the formation of SARS-CoV-2 homo-dimer and the interaction with Glu166 may lead to the formation of inactive monomer, which will affect the enzyme activity of Mpro [49]. All three compounds interacted with His41, Cys145 and Glu166, which are similar to PF-07321332 [50] and α-ketoamide that were previously reported to have inhibitory effects on Mpro. For PLpro, all three compounds form hydrogen bonding interactions with Cys111. Interestingly, compound A3659 also forms a hydrogen bonding interaction with His272 in the catalytic triad. In addition, the three compounds also interact with other amino acid residues within PLpro cavity, such as Asn109, Asn110, Tyr112 and Tyr273, through hydrogen bonds and π–π interactions. On the basis of the preceding analysis, all three compounds interact with amino acid residues in the catalytic dyad of Mpro (His41, Cys145) and the catalytic triad of PLpro (Cys111, His272, Asp286). The benzene or naphthalene ring of A3175, A3659 and A3777 occupy the pocket where His41 is located of Mpro, and all three interact with key amino acid residues such as Cys145 and Glu166 through the carbonyl group on the backbone [50]. Previous studies found that the BL2 loop (residues 265–271) of the PLpro protein had a role in substrate recognition. The peptide inhibitor VIR251 expands the BL2 loop [51] and the small molecule inhibitor GRL0617 closes the BL2 loop [52]. Compared with GRL0617, compounds A3175, A3659 and A3777 all approach the BL2 loop from the other direction. This pose may affect the binding of PLpro to the substrate and will be further investigated in dynamics simulations.

### Dynamics simulations results

**Figure 7.**
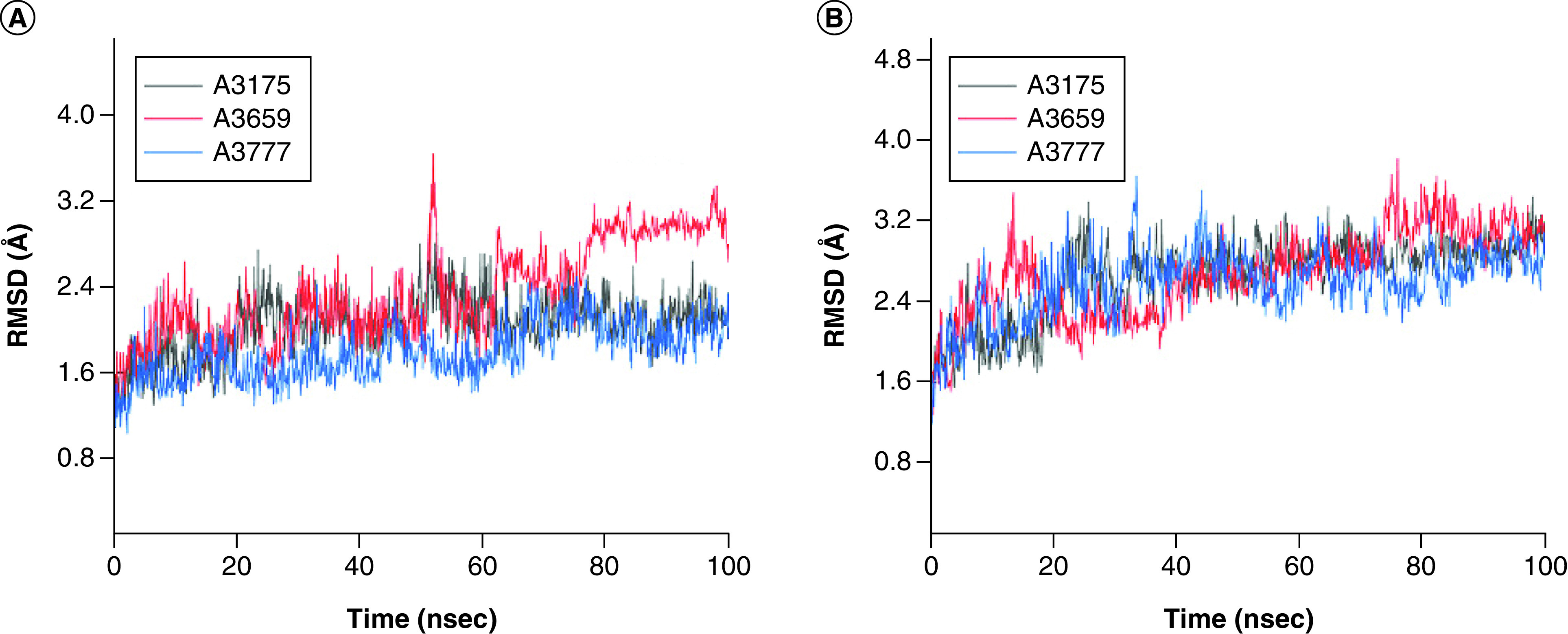
Root mean square deviation of protein backbone atoms. **(A)** Main protease and **(B)** papain-like protease. RMSD: Root mean square deviation.

**Figure 8.**
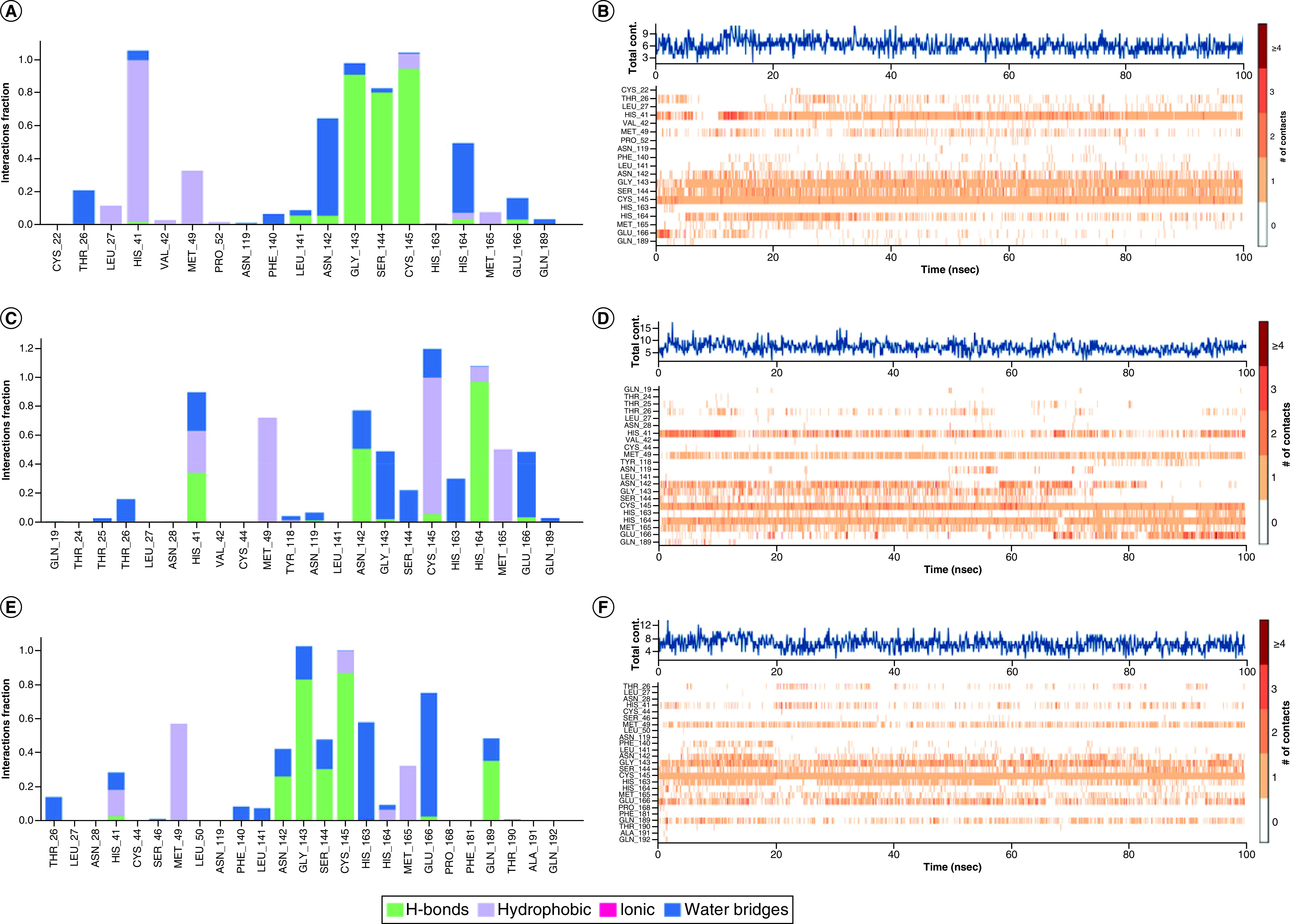
The interaction and counts for main protease protein with ligands during dynamics simulations. **(A)** The interaction of A3175 and main protease (Mpro) protein. **(B)** The counts of A3175 and Mpro protein interactions. **(C)** The interaction of A3659 and Mpro protein. **(D)** The counts of A3659 and Mpro protein interactions. **(E)** The interaction of A3777 and Mpro protein. **(F)** The counts of A3777 and Mpro protein interactions.

**Figure 9.**
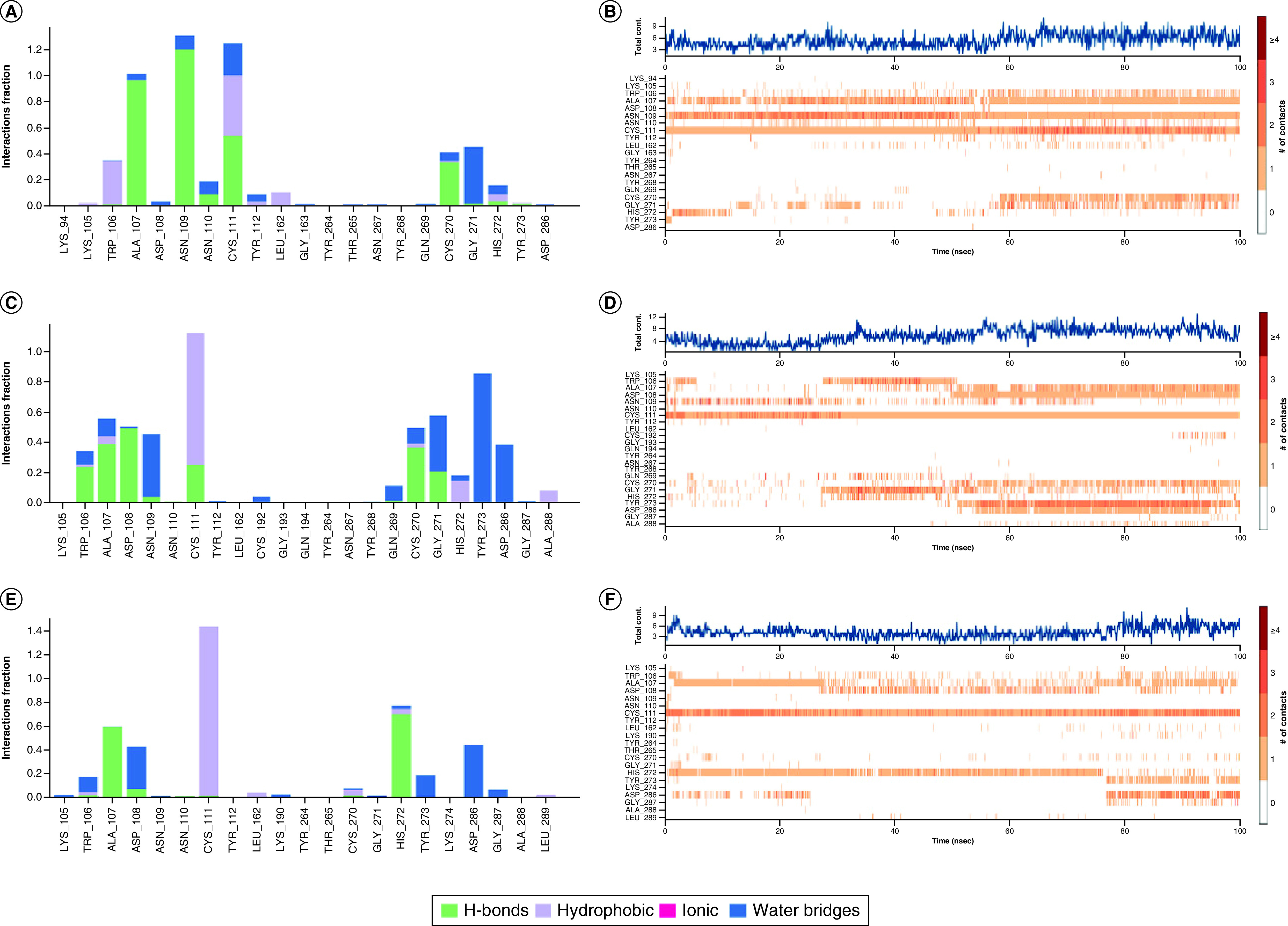
The interaction and counts for papain-like protease protein with ligands during dynamics simulations. **(A)** The interaction of A3175 and papain-like protease (PLpro) protein. **(B)** The counts of A3175 and PLpro protein interactions. **(C)** The interaction of A3659 and PLpro protein. **(D)** The counts of A3659 and PLpro protein interactions. **(E)** The interaction of A3777 and PLpro protein. **(F)** The counts of A3777 and PLpro protein interactions.

The results of molecular dynamics simulations of the complexes of A3175, A3659 and A3777 with Mpro and PLpro are shown in Figures 7–9. As shown in Figure 7A, the root mean square deviation (RMSD) of the A3175-Mpro system and the A3777-Mpro system reached stability after 20 ns, and the RMSD of these two systems stabilized at 1.7–3.0 Å and 1.3–2.5 Å, respectively. The RMSD of the A3659-Mpro system fluctuated greater than the other two systems, finally stabilizing at 1.7–3.3 Å. As shown in Figure 7B, all compound-PLpro systems reached equilibrium after 40 ns, and the RMSD values for these three systems were stable at 2.3–3.4 Å, 2.2–3.8 Å and 2.2–3.5 Å, respectively. The amplitudes of RMSD for all six complex systems were within 2 Å after equilibrium, indicating that all six complex systems were stable.

The results of the protein–ligand interactions during the dynamics simulations are shown in Figures 8 and 9. For Mpro, A3175 and A3777 have excellent hydrogen bond occupancy with Cys145 (A3175: 94.5%; A3777: 86.8%) and A3659 interacts with Cys145 mainly by hydrophobic interactions. At the same time, these three compounds are also in contact with His41 through hydrogen bonding, hydrophobic interactions and water bridge interactions. This demonstrated that all three compounds effectively occupy the catalytic dyad of the Mpro and prevent its substrate from binding to the Mpro. A recent study showed that PF-07321332 and α-ketoamide performed better than Lopinavir and Ritonavir, due to their ability to disrupt the catalytic dyad residue His41-Cys145 of Mpro [53]. These three compounds disrupt the catalytic dyad by interacting with His41-Cys145, which is similar to PF-07321332 and α-ketoamide. Also, these three compounds form frequent interactions with other amino acid residues within the Mpro cavity, such as Met49 Gly143 and Glu166. A3659 and A3777 formed stable interactions with Glu166 via the water bridge (A3659: 45%; A3777: 73%), indicating that they could disrupt the dimerization of Mpro and affect its catalytic activity. This shows that all three compounds could stably occupy the Mpro cavity and block the binding of the substrate. For PLpro, all three compounds are frequently in contact with Cys111, His272, Asp286 in the catalytic site via hydrogen bonding, hydrophobic interactions and water bridge interactions and form interactions with other amino acid residues in the cavity (e.g., Ala107, Asn109 and Cys270). This suggests that these three compounds were solidly bound to the PLpro cavity. Interestingly, all three compounds were observed to interact with amino acids on the BL2 loop (e.g., Gln269, Cys270). Moreover, the alteration of the BL2 loop affects the binding of PLpro to its substrate, which subsequently influences the replication of the virus.

## Conclusion

For the design of dual-target inhibitors, the balancing of the effects between the two targets was a complicated and challenging task. DL has unique advantages in balancing multitarget activity. In this work, the dual-target covalent inhibitors of Mpro and PLpro were designed by deep RL and virtual screening. First, a novel library of small molecule inhibitors for SARS-CoV-2 was generated using deep RL. The GSMs inherited the features of the small molecules in the training set. A total of 4428 small molecule compounds were obtained. Subsequently, covalent docking studies were carried out on these small molecules. One hundred five small molecules that can covalently bind to cysteine at the active site of Mpro and PLpro were obtained. The top three compounds according to overall scores were analyzed for docking results. All three compounds can form covalent bonds with cysteines in the catalytic sites of Mpro and PLpro proteins. In addition, the interaction of compounds with key amino acid residues within the cavity of Mpro (His41, Cys145) and PLpro (Cys111, His272) proteins was also observed. Dynamics simulations were then carried out for the three compounds. The RMSD results show that all three compounds can bind stably to Mpro and PLpro proteins. The analysis of compound–protein interactions during dynamics simulations showed that all three compounds formed stable hydrogen bonding interactions or hydrophobic interactions with key amino acid residues within the protein cavity, such as His41, Cys145 and Cys111. To summarize, the three compounds have the potential to be lead compounds for dual-target inhibitors of SARS-CoV-2 Mpro and PLpro. This may provide a good foundation for the discovery of anti-SARS-CoV-2 drugs. This study applied deep RL to the design of covalent inhibitors and produced positive results.

## Future perspective

Dual-targeted drugs have the advantage of fewer adverse effects and better therapeutic outcomes than single-targeted drugs. However, most of the current multitargeted drugs have been discovered by chance, and the rational design of multi-targeted drugs is a complex and sophisticated task. One of the difficulties in the design of dual-target inhibitors is balancing the activity of the compound between the two targets. The recent rise in artificial intelligence drug design offers a new option. Our method incorporates the advantages of existing drug design techniques with artificial intelligence technologies, which will accelerate the discovery of and optimize drug lead compounds, as well as reduce the cost of drug development.

**Summary points**

- Applying deep reinforcement learning to the design of covalent inhibitors.
- Deep reinforcement learning methods were used to learn the features of existing main protease (Mpro) and papain-like protease (PLpro) inhibitors.
- A range of small molecules with the features of Mpro and PLpro inhibitors were generated.
- The generated small molecules were screened using covalent docking.
- Molecular dynamics simulations further investigate the stability of small molecules bound to proteins and their interactions.
- High frequency interactions of selected compounds with key residues at the binding site were observed.
- Selected compounds can facilitate the rational design of anti-SARS-CoV-2 drugs.
