# Supplementary material for: Design of SARS-CoV-2 Mpro, PLpro dual-target inhibitors based on deep reinforcement learning and virtual screening
Source: Future Med Chem. 2022 Feb 27:10.4155/fmc-2021-0269. doi: 10.4155/fmc-2021-0269 (PMC8920029; doi:10.4155/fmc-2021-0269)
Supplement: Supplementary file 1 [file Supplementary-Material.docx]

**Table S1.** Docking scores and MM-GBSA scores of the top 20 compounds that bind the two proteins (kcal/mol).

| **Compound ID** | **Mpro** | | **PLpro** | |
| --- | --- | --- | --- | --- |
|  | **MMGBSA dG Bind** | **cdock affinity** | **MMGBSA dG Bind** | **cdock affinity** |
| A3777 | -84.01 | -6.013389 | -60.3 | -6.20462 |
| A3175 | -78.82 | -8.173824 | -59.86 | -5.67662 |
| A3659 | -79.2 | -7.595511 | -51.23 | -5.50407 |
| A4248 | -88.65 | -7.966509 | -37.03 | -5.44091 |
| A4421 | -75.2 | -6.67752 | -52.99 | -5.23546 |
| A3268 | -84.14 | -7.754545 | -54.93 | -5.00975 |
| A4215 | -104.65 | -8.130415 | -54.99 | -4.66809 |
| A4305 | -91.51 | -7.887369 | -55.52 | -3.93955 |
| A2834 | -66.59 | -7.056858 | -32.64 | -3.90176 |
| A4298 | -72.06 | -6.997877 | -56.69 | -3.72773 |
| A4288 | -94.84 | -9.647889 | -56.4 | -3.33339 |
| A4111 | -100.3 | -9.88509 | -49.52 | -3.19286 |
| A3769 | -72.31 | -7.349149 | -36.47 | -3.14197 |
| A4331 | -89.96 | -7.139248 | -54.06 | -3.12894 |
| A3495 | -84.47 | -7.896126 | -44.83 | -3.02782 |
| A4335 | -79.37 | -8.824667 | -49.01 | -2.94751 |
| A4071 | -70.66 | -7.33582 | -43.82 | -2.11438 |
| A4295 | -109.53 | -9.393829 | -53.31 | -1.22277 |
| A3316 | -78.24 | -6.868644 | -37.89 | 0.31764 |
| A4371 | -92.21 | -6.080561 | -42.37 | 51.92696 |





**Figure S1.** The structure of top20 compounds.
